# Analysis of early postoperative risk factors for hypopituitarism after endoscopic endonasal transsphenoidal surgery for pituitary neuroendocrine tumors

**DOI:** 10.1097/MD.0000000000050217

**Published:** 2026-08-14

**Authors:** Li Zhang, Qiuyan Gao, Wei Dong, Yu Zhang, Xiao Liu Dong

**Affiliations:** a Department of Neurosurgery, Tangshan People’s Hospital, Tangshan City, Hebei Province, China; b Physical Examination Center, Tangshan People’s Hospital, Tangshan City, Hebei Province, China; c Department of Critical Care Medicine, Tangshan People’s Hospital, Tangshan City, Hebei Province, China; d Department of Neurology, Tangshan People’s Hospital, Tangshan City, Hebei Province, China.

**Keywords:** endoscopic endonasal transsphenoidal surgery, gross total resection, hypopituitarism, Knosp grade, pituitary neuroendocrine tumor, risk factors

## Abstract

Pituitary neuroendocrine tumors (PitNETs) are commonly managed with endoscopic endonasal transsphenoidal surgery (EETS), but early postoperative hypopituitarism remains a clinically important complication. This retrospective cohort study analyzed early postoperative risk factors for hypopituitarism in 124 patients with PitNETs who underwent EETS at a single center between January 2022 and December 2024. Endocrine status was assessed preoperatively, during the immediate postoperative period before discharge (postoperative days 3–7), and at scheduled outpatient reassessments at 1 and 3 months after surgery; the mean interval from surgery to endpoint endocrine assessment was 3.2 ± 0.6 months. Patients were categorized into a hypopituitarism group (n = 38) and a non-hypopituitarism group (n = 86) based on postoperative endocrine evaluation across the hypothalamic–pituitary–adrenal, thyroid, gonadal, and growth hormone axes. Demographic, radiologic, perioperative, and endocrine data were collected, and univariate and multivariate logistic regression analyses were performed. Patients who developed hypopituitarism were older and had larger tumors, higher Knosp grades, more frequent suprasellar extension, longer operation times, greater intraoperative blood loss, more intraoperative cerebrospinal fluid leakage, and lower rates of gross total resection. In the multivariate model, maximum tumor diameter (adjusted odds ratio [aOR] per 10-mm increase = 2.02, 95% confidence interval [CI]: 1.09–3.74) and Knosp grade 3–4 (aOR = 2.37, 95% CI: 1.03–5.46) were identified as independent risk factors for early postoperative hypopituitarism, whereas gross total resection was independently associated with a reduced risk (aOR = 0.39, 95% CI: 0.16–0.96). These findings indicate that tumor anatomy and extent of resection are key determinants of early endocrine outcomes after EETS and should be incorporated into preoperative risk stratification and postoperative endocrine surveillance; however, they should not be extrapolated to long-term pituitary functional recovery or deterioration without further follow-up.

## 1. Introduction

Pituitary neuroendocrine tumors (PitNETs) are common intracranial neoplasms arising from adenohypophyseal cells and account for a substantial proportion of primary brain tumors. Recent revisions of the World Health Organization classification have consolidated the concept of PitNETs and emphasized their heterogeneous biological behavior, hormonal activity, and potential for local invasion and recurrence.^[1,2]^ These changes have highlighted the need to integrate endocrine, radiologic, and histopathologic features when evaluating treatment strategies and long-term outcomes in patients with PitNETs.^[3]^ Endoscopic endonasal transsphenoidal surgery (EETS) has become the preferred primary treatment for most PitNETs because it provides panoramic visualization, improved access to the suprasellar and parasellar regions, and favorable rates of tumor control. Large contemporary series and learning-curve analyses have shown that the endoscopic endonasal approach can achieve high rates of gross total resection (GTR) with acceptable morbidity when performed in experienced centers.^[4,5]^ Nevertheless, endocrine complications remain a major concern. Postoperative anterior pituitary insufficiency and diabetes insipidus are among the most frequent adverse events after endoscopic transsphenoidal surgery and can lead to long-term dependence on hormone replacement therapy and reduced quality of life.^[6,7]^

Recent observational studies have described early postoperative complication profiles after endoscopic endonasal surgery, including hypopituitarism, hyponatremia, cerebrospinal fluid (CSF) leak, and infection, and have proposed risk factors such as larger tumor size, cavernous sinus invasion, and surgical complexity. However, most available reports have focused on global complication rates or specific endpoints such as delayed hyponatremia, rather than systematically evaluating determinants of postoperative pituitary axis dysfunction.^[8]^ Furthermore, many cohorts combine microscopic and endoscopic techniques or include heterogeneous sellar pathologies, which limits the ability to isolate risk factors that are specific to PitNETs treated via a standardized endoscopic approach. At the same time, emerging prediction models and machine-learning approaches underscore that tumor diameter, invasion into the cavernous sinus, surgical trauma, and residual tumor are important determinants of endocrine prognosis, but these models are still in the early stages of validation and often do not focus specifically on hypopituitarism as a primary endpoint.^[9,10]^ There remains a need for methodologically consistent studies that examine how patient characteristics, radiologic markers of invasiveness, and intraoperative factors jointly influence the risk of postoperative hypopituitarism in the modern PitNET framework.

Against this background, the present study analyzes clinical, imaging, and perioperative variables in a consecutive cohort of patients with PitNETs undergoing EETS by a single experienced team. By characterizing preoperative pituitary axis status and postoperative endocrine outcomes and by applying multivariable logistic regression, this work aims to clarify which anatomical and surgical factors are independently associated with hypopituitarism after surgery and to provide evidence that may support risk stratification and individualized perioperative endocrine management in PitNET patients.

## 2. Methods

### 2.1. Study design

This retrospective cohort study consecutively enrolled patients with PitNETs who underwent EETS at our institution between January 2022 and December 2024. The endpoint was defined as early postoperative hypopituitarism and was determined within the first 3 months after surgery. The mean follow-up duration for endocrine endpoint ascertainment was 3.2 ± 0.6 months. Postoperative hypopituitarism was defined according to standardized endocrine assessment, and patients were categorized into a hypopituitarism group and a non-hypopituitarism group based on whether clinically and biochemically confirmed new-onset or worsened hypopituitarism occurred after surgery. Inclusion criteria were pathologically confirmed PitNET; receipt of EETS as the primary surgical approach during the study period; availability of complete preoperative clinical, imaging, and laboratory data; documented preoperative baseline pituitary hormone evaluation and at least one standardized postoperative endocrine reassessment within the early postoperative follow-up window; and sufficient clinical follow-up to ascertain postoperative pituitary function. Exclusion criteria were prior pituitary surgery, radiotherapy, or medical interventions that could confound postoperative endocrine outcomes; coexisting sellar/parasellar lesions other than PitNETs (e.g., craniopharyngioma and Rathke cleft cyst); severe preoperative hypopituitarism, defined as panhypopituitarism or deficiency of 2 or more anterior pituitary axes requiring hormone replacement, or cases in which new-onset or worsened postoperative dysfunction could not be distinguished from preexisting dysfunction; incomplete key perioperative records or missing hormone data; and severe systemic illness affecting hormonal assessment. Patients with isolated preoperative single-axis abnormalities were not excluded because postoperative endocrine change could be evaluated by comparison with each patient’s own baseline. These patients were retained in the cohort; stable preexisting single-axis abnormalities were not counted as postoperative events, and baseline dysfunction was recorded as preoperative any-axis hypopituitarism for statistical analysis. The study was conducted in accordance with the Declaration of Helsinki and was approved by the institutional medical ethics committee. Written informed consent was obtained from all participants prior to inclusion in the analysis.

### 2.2. Surgical protocol

All patients underwent standardized endoscopic EETS following a uniform institutional protocol, and all procedures were performed by the same dedicated pituitary surgical team with consistent perioperative workflows. Under general anesthesia, patients were positioned supine with the head slightly extended and secured; neuronavigation was used when indicated. A binostril endoscopic approach was adopted in most cases. After routine nasal cavity preparation, the sphenoid sinus was accessed, and the sellar floor was exposed. The sellar bone was then opened, and the dura was incised under endoscopic visualization. Tumor resection was performed using suction and curettes, with careful technique to maximize safe resection while minimizing traction on normal pituitary tissue and the stalk. Hemostasis was achieved using standard intraoperative measures. Postoperatively, patients were managed with a standardized pathway including neurological monitoring, fluid and electrolyte surveillance, assessment for diabetes insipidus and CSF rhinorrhea, and scheduled endocrine testing to evaluate pituitary axis function.

### 2.3. Diagnostic criteria for postoperative hypopituitarism

Postoperative hypopituitarism was defined as new-onset or worsened deficiency of 1 or more anterior pituitary axes after EETS, confirmed by postoperative endocrine testing compared with preoperative baseline, with findings consistent with central (pituitary) dysfunction. Specifically, this included: secondary adrenal insufficiency (inadequate morning cortisol and/or abnormal confirmatory testing), central hypothyroidism (low free thyroxine with low/inappropriately normal thyroid-stimulating hormone), central hypogonadism (low sex hormones with low/inappropriately normal luteinizing hormone/follicle-stimulating hormone), and possible growth hormone deficiency (low age-adjusted insulin-like growth factor 1 with confirmatory stimulation testing when performed). Patients were categorized as having hypopituitarism if at least 1 axis met these criteria. In patients with an isolated preoperative single-axis abnormality, only a new deficiency in another axis or clinically/biochemically worsened deficiency requiring new initiation or escalation of hormone replacement was classified as postoperative hypopituitarism. Stable preexisting isolated abnormalities were not counted as new postoperative events.

### 2.4. Data collection

Clinical data were collected retrospectively from the institutional electronic medical record, radiology archive, and laboratory information systems for all eligible patients. Demographic variables included age, sex, and body mass index (BMI) at the time of surgery. Baseline clinical information comprised vascular and metabolic comorbidities (hypertension, diabetes mellitus, coronary artery disease) and a history of long-term systemic glucocorticoid therapy, defined as continuous use for ≥3 months before surgery. Tumor-related radiologic variables were extracted from preoperative magnetic resonance imaging reports and, when necessary, reviewed by a neurosurgeon and neuroradiologist. Recorded parameters included maximum tumor diameter, tumor size category (micro-, macro-, or giant PitNET), Knosp grade (0–4), presence of suprasellar extension, parasellar extension, and optic chiasm compression. Tumors were classified as functional or nonfunctional according to preoperative endocrine evaluation and clinical presentation.

Perioperative surgical variables were obtained from operative notes and anesthesia records. These included operation time, estimated intraoperative blood loss, use of neuronavigation, occurrence of intraoperative CSF leak, and skull base reconstruction strategy. The extent of resection was categorized as GTR or non-GTR based on early postoperative magnetic resonance imaging and operative documentation. Early postoperative complications recorded during the index hospitalization included transient or persistent diabetes insipidus, early hyponatremia, postoperative CSF leak, and intracranial or systemic infection. Postoperative pituitary function was assessed using the same axis-based framework at routine postoperative endocrine evaluations during follow-up. Postoperative hypopituitarism status (yes/no) was determined by comparing postoperative endocrine findings with the preoperative baseline according to the predefined diagnostic criteria. All collected data were anonymized before analysis.

Baseline endocrine testing was performed within 1 week before surgery. Postoperative endocrine evaluations were scheduled in the immediate postoperative period before discharge (postoperative days 3–7), at 1 month after surgery, and at 3 months after surgery. The primary analysis used the 3-month endocrine assessment when available or the latest standardized assessment within the predefined early postoperative window. Additional hormone testing was performed when symptoms, electrolyte abnormalities, or replacement therapy adjustments indicated possible pituitary axis dysfunction. Therefore, the present results primarily reflect early postoperative endocrine risk rather than long-term pituitary function.

### 2.5. Statistical analysis

All statistical analyses were performed using IBM SPSS Statistics, version 26.0 (IBM Corp., Armonk). Continuous variables were summarized as means ± standard deviations and compared between the hypopituitarism and non-hypopituitarism groups using independent-samples *t* tests. Categorical variables were presented as counts and percentages and compared using Pearson chi-square (χ^2^) tests. Univariate logistic regression analyses were conducted with postoperative hypopituitarism (yes/no) as the dependent variable to estimate odds ratios (ORs) and corresponding 95% confidence intervals (CIs) for potential risk factors. Variables with a *P*-value < .10 in univariate analysis, together with clinically relevant covariates, were entered into a multivariate logistic regression model to identify independent predictors of postoperative hypopituitarism. A stepwise selection procedure was applied to derive the final model. Multicollinearity among covariates was assessed using variance inflation factors. All statistical tests were two-sided, and a *P*-value < .05 was considered statistically significant.

## 3. Results

### 3.1. Baseline demographic and clinical characteristics

A total of 124 patients were included in the analysis, comprising 38 patients in the hypopituitarism group (30.6%) and 86 patients in the non-hypopituitarism group (69.4%). Baseline demographic and clinical characteristics were generally comparable between the 2 groups. All included patients completed at least 1 standardized endocrine reassessment within the predefined early postoperative follow-up window; the mean interval from surgery to endpoint endocrine assessment was 3.2 ± 0.6 months. Patients who developed postoperative hypopituitarism were significantly older than those without hypopituitarism (53.0 ± 9.6 vs 48.7 ± 10.2 years, *t* = 2.20, *P* = .029). The proportion of male patients did not differ significantly between groups (57.9% vs 46.5%, χ^2^ = 1.37, *P* = .243), and BMI was similar (24.4 ± 2.7 vs 24.2 ± 2.9 kg/m^2^, *t* = 0.36, *P* = .718). With respect to comorbidities, the hypopituitarism group showed numerically higher rates of hypertension (47.4% vs 30.2%, χ^2^ = 3.38, *P* = .066), diabetes mellitus (26.3% vs 16.3%, χ^2^ = 1.70, *P* = .192), and coronary artery disease (18.4% vs 8.1%, χ^2^ = 2.78, *P* = .095), although these differences did not reach statistical significance. Notably, a history of long-term systemic glucocorticoid therapy (≥3 months before surgery) was more frequent in the hypopituitarism group (21.1% vs 8.1%), and this difference was statistically significant (χ^2^ = 4.13, *P* = .042; Table 1).

**Table 1:** Baseline demographic and clinical characteristics of patients with and without postoperative hypopituitarism.

Variable	Hypopituitarism group (n = 38)	Non-hypopituitarism group (n = 86)	Test statistic	*P*-value
Age, yr	53.0 ± 9.6	48.7 ± 10.2	*t* = 2.20	.029
Male sex, n (%)	22 (57.9)	40 (46.5)	χ^2^ = 1.37	.243
BMI, kg/m^2^	24.4 ± 2.7	24.2 ± 2.9	*t* = 0.36	.718
Hypertension, n (%)	18 (47.4)	26 (30.2)	χ^2^ = 3.38	.066
Diabetes mellitus, n (%)	10 (26.3)	14 (16.3)	χ^2^ = 1.70	.192
Coronary artery disease, n (%)	7 (18.4)	7 (8.1)	χ^2^ = 2.78	.095
Long-term glucocorticoid therapy, n (%)*	8 (21.1)	7 (8.1)	χ^2^ = 4.13	.042

BMI = body mass index.

* Long-term glucocorticoid therapy was defined as systemic glucocorticoid use for ≥3 months before surgery.

### 3.2. Tumor-related radiologic features and perioperative surgical characteristics

The maximum tumor diameter was significantly larger in the hypopituitarism group than in the non-hypopituitarism group (28.5 ± 7.2 vs 23.6 ± 6.8 mm, *t* = 3.55, *P* = .001). Although the distribution of microadenomas, macroadenomas, and giant adenomas showed a higher proportion of giant adenomas in the hypopituitarism group compared with the non-hypopituitarism group (31.6% vs 16.3%), the overall difference in tumor size categories did not reach statistical significance (χ^2^ = 4.65, *P* = .098). Markers of invasive growth and anatomical extension were more frequent among patients who developed postoperative hypopituitarism. High Knosp grade (3–4) was significantly more common in the hypopituitarism group than in the non-hypopituitarism group (63.2% vs 37.2%, χ^2^ = 7.17, *P* = .007). Similarly, suprasellar extension (78.9% vs 55.8%, χ^2^ = 6.04, *P* = .014) and optic chiasm compression (57.9% vs 34.9%, χ^2^ = 5.73, *P* = .017) were more frequently observed in the hypopituitarism group. The proportion of functional tumors was comparable between groups (52.6% vs 58.1%, χ^2^ = 0.33, *P* = .568). Regarding perioperative parameters, operation time was significantly longer in the hypopituitarism group compared with the non-hypopituitarism group (155 ± 32 vs 140 ± 30 minutes, *t* = 2.45, *P* = .017), and estimated intraoperative blood loss was higher (220 ± 90 vs 185 ± 80 mL, *t* = 2.06, *P* = .043). The use of neuronavigation was similar between the 2 groups (78.9% vs 83.7%, χ^2^ = 0.41, *P* = .521). Intraoperative CSF leakage occurred more often in the hypopituitarism group than in the non-hypopituitarism group (47.4% vs 29.1%, χ^2^ = 3.90, *P* = .048). GTR was achieved less frequently in the hypopituitarism group (63.2% vs 83.7%, χ^2^ = 6.37, *P* = .012). Early postoperative complications showed a higher rate of transient diabetes insipidus in the hypopituitarism group (42.1% vs 20.9%, χ^2^ = 5.94, *P* = .015), whereas early hyponatremia exhibited a nonsignificant trend toward a higher incidence (26.3% vs 12.8%, χ^2^ = 3.43, *P* = .064). Early postoperative CSF leak and infection rates were low and did not differ significantly between groups (Table 2).

**Table 2:** Tumor-related radiologic features and perioperative surgical characteristics in patients with and without postoperative hypopituitarism.

Variable	Hypopituitarism group (n = 38)	Non-hypopituitarism group (n = 86)	Test statistic	*P*-value
Tumor maximum diameter, mm, mean ± SD	28.5 ± 7.2	23.6 ± 6.8	*t* = 3.55	.001
Tumor size category, n (%)	Micro: 4 (10.5); macro: 22 (57.9); giant: 12 (31.6)	Micro: 18 (20.9); macro: 54 (62.8); giant: 14 (16.3)	χ^2^ = 4.65 (df = 2)	.098
Knosp grade 3–4, n (%)	24 (63.2)	32 (37.2)	χ^2^ = 7.17	.007
Suprasellar extension, n (%)	30 (78.9)	48 (55.8)	χ^2^ = 6.04	.014
Optic chiasm compression, n (%)	22 (57.9)	30 (34.9)	χ^2^ = 5.73	.017
Functional tumor, n (%)	20 (52.6)	50 (58.1)	χ^2^ = 0.33	.568
Operation time, min, mean ± SD	155 ± 32	140 ± 30	*t* = 2.45	.017
Estimated blood loss, mL, mean ± SD	220 ± 90	185 ± 80	*t* = 2.06	.043
Neuronavigation used, n (%)	30 (78.9)	72 (83.7)	χ^2^ = 0.41	.521
Intraoperative CSF leak, n (%)	18 (47.4)	25 (29.1)	χ^2^ = 3.90	.048
Gross total resection (GTR), n (%)	24 (63.2)	72 (83.7)	χ^2^ = 6.37	.012
Transient diabetes insipidus (DI), n (%)	16 (42.1)	18 (20.9)	χ^2^ = 5.94	.015
Early hyponatremia, n (%)	10 (26.3)	11 (12.8)	χ^2^ = 3.43	.064
Early postoperative CSF leak, n (%)	4 (10.5)	3 (3.5)	χ^2^ = 2.45	.117
Early postoperative infection, n (%)	2 (5.3)	2 (2.3)	χ^2^ = 0.73	.393

CSF = cerebrospinal fluid, SD = standard deviation.

### 3.3. Univariate analysis of potential risk factors for postoperative hypopituitarism

Univariate logistic regression was performed with postoperative hypopituitarism as the dependent variable. Among continuous variables, tumor maximum diameter and operation time were significantly associated with postoperative hypopituitarism. Each 10-mm increase in tumor maximum diameter was associated with higher odds of hypopituitarism (OR = 2.78, 95% CI: 1.46–5.30, *P* = .002). Each 30-minute increase in operation time was also associated with an increased risk (OR = 1.58, 95% CI: 1.11–2.25, *P* = .011). Age (per 10-year increase) and estimated blood loss (per 50-mL increase) showed a nonsignificant trend toward higher odds of hypopituitarism (OR = 1.36, 95% CI: 0.89–2.06, *P* = .155; OR = 1.27, 95% CI: 0.99–1.62, *P* = .061, respectively), whereas BMI was not associated with the outcome. For categorical variables, long-term glucocorticoid therapy was significantly associated with postoperative hypopituitarism (OR = 3.01, 95% CI: 1.00–9.02, *P* = .049). Markers of local invasiveness and anatomical extension were also related to hypopituitarism: Knosp grade 3 to 4 (vs 0–2; OR = 2.89, 95% CI: 1.31–6.38, *P* = .008), suprasellar extension (OR = 2.97, 95% CI: 1.22–7.22, *P* = .016), and optic chiasm compression (OR = 2.57, 95% CI: 1.17–5.61, *P* = .018) were all significant risk factors. Intraoperative CSF leak demonstrated a borderline association (OR = 2.20, 95% CI: 1.00–4.83, *P* = .051). GTR appeared protective, with lower odds of hypopituitarism compared with non-GTR (OR = 0.33, 95% CI: 0.14–0.80, *P* = .014). Sex, functional status of the tumor, hypertension, diabetes mellitus, coronary artery disease, and preoperative any-axis hypopituitarism were not significantly associated with postoperative hypopituitarism in univariate analysis (Table 3).

**Table 3:** Univariate logistic regression analysis of potential risk factors for postoperative hypopituitarism.

Variable	OR	95% CI	*P*-value
Age, per 10-yr increase	1.36	0.89–2.06	.155
BMI, per 1 kg/m^2^ increase	0.98	0.85–1.12	.747
Tumor maximum diameter, per 10-mm increase	2.78	1.46–5.30	.002
Operation time, per 30-min increase	1.58	1.11–2.25	.011
Estimated blood loss, per 50-mL increase	1.27	0.99–1.62	.061
Male sex (vs female)	1.58	0.73–3.42	.244
Hypertension (yes vs no)	2.08	0.95–4.56	.068
Diabetes mellitus (yes vs no)	1.84	0.73–4.62	.196
Coronary artery disease (yes vs no)	2.55	0.83–7.87	.104
Long-term glucocorticoid therapy (yes vs no)	3.01	1.00–9.02	.049
Functional tumor (yes vs nonfunctional)	0.80	0.37–1.72	.569
Knosp grade 3–4 (vs 0–2)	2.89	1.31–6.38	.008
Suprasellar extension (yes vs no)	2.97	1.22–7.22	.016
Optic chiasm compression (yes vs no)	2.57	1.17–5.61	.018
Intraoperative CSF leak (yes vs no)	2.20	1.00–4.83	.051
Gross total resection (vs non-GTR)	0.33	0.14–0.80	.014
Preoperative any-axis hypopituitarism (yes vs no)	1.61	0.70–3.70	.262

BMI = body mass index, CI = confidence interval, CSF = cerebrospinal fluid, GTR = gross total resection, OR = odds ratio.

### 3.4. Multivariate logistic regression for independent risk factors of postoperative hypopituitarism

Variables with a *P*-value < .10 in univariate analysis (tumor maximum diameter, operation time, estimated blood loss, hypertension, long-term glucocorticoid therapy, Knosp grade 3 to 4, suprasellar extension, optic chiasm compression, intraoperative CSF leak, and GTR) and those considered clinically relevant (age) were entered into a multivariate logistic regression model. To reduce collinearity, operation time, estimated blood loss, and optic chiasm compression were not retained in the final model after stepwise selection, and tumor maximum diameter (continuous), Knosp grade, suprasellar extension, intraoperative CSF leak, long-term glucocorticoid therapy, GTR, and age were preserved. Variance inflation factors for all included variables were <2.0, indicating no relevant multicollinearity. The adjusted results are shown in Table 4. After adjustment, larger tumor size remained independently associated with a higher risk of postoperative hypopituitarism (adjusted OR [aOR] per 10-mm increase = 2.02, 95% CI: 1.09–3.74, *P* = .025). Knosp grade 3 to 4 (vs 0–2) was also an independent risk factor (aOR = 2.37, 95% CI: 1.03–5.46, *P* = .042). GTR was independently associated with a lower risk of postoperative hypopituitarism (aOR = 0.39, 95% CI: 0.16–0.96, *P* = .040), suggesting a protective effect. Age, long-term glucocorticoid therapy, suprasellar extension, and intraoperative CSF leak did not remain statistically significant after multivariable adjustment, although their point estimates indicated trends consistent with the univariate analysis (Table 4).

**Table 4:** Multivariate logistic regression analysis of independent risk factors for postoperative hypopituitarism.

Variable	aOR	95% CI	*P*-value
Age, per 10-yr increase	1.18	0.77–1.82	.449
Tumor maximum diameter, per 10-mm increase	2.02	1.09–3.74	.025
Long-term glucocorticoid therapy (yes vs no)	2.21	0.69–7.06	.181
Knosp grade 3–4 (vs 0–2)	2.37	1.03–5.46	.042
Suprasellar extension (yes vs no)	1.52	0.60–3.88	.378
Intraoperative CSF leak (yes vs no)	1.94	0.84–4.50	.121
Gross total resection (vs non-GTR)	0.39	0.16–0.96	.040

aOR = adjusted odds ratio, CI = confidence interval, CSF = cerebrospinal fluid, GTR = gross total resection.

## 4. Discussion

This study systematically evaluated risk factors for postoperative hypopituitarism in patients with pituitary neuroendocrine tumors (PitNETs) undergoing EETS. Postoperative hypopituitarism occurred in approximately one-third of patients, indicating that endocrine deterioration remains a frequent adverse outcome despite advances in endoscopic techniques. In univariate analyses, older age, larger tumor size, parameters reflecting local invasiveness (higher Knosp grade, suprasellar extension, optic chiasm compression), longer operative time, greater intraoperative blood loss, intraoperative CSF leakage, long-term glucocorticoid exposure, and non-GTR were associated with postoperative hypopituitarism to varying degrees. After multivariable adjustment, 3 variables remained independently associated with the endpoint: larger maximum tumor diameter and Knosp grade 3 to 4 emerged as independent risk factors, whereas GTR was independently associated with a lower risk of hypopituitarism.

The association between tumor size and early endocrine impairment is clinically plausible. Larger lesions are more likely to compress and distort the normal gland and stalk, compromise the pituitary portal circulation, and reduce functional reserve before surgery. They also usually require broader decompression and greater manipulation during tumor removal. Similarly, Knosp grade 3 to 4 reflects cavernous sinus invasion and a more complex tumor–gland interface. In such cases, clear pseudocapsular dissection may be difficult, and the surgeon must balance endocrine preservation, vascular safety, and extent of resection. These anatomical features explain why tumor diameter and high Knosp grade retained independent associations after adjustment for perioperative complexity.^[11,12]^

An important observation in this series is that GTR was independently associated with a reduced risk of postoperative hypopituitarism, even after adjustment for tumor size and Knosp grade. This association should not be understood as a simple volume–outcome effect in which “more resection” directly protects endocrine function. Rather, GTR in the endoscopic era is often achieved in cases with more favorable anatomy and clearer tumor–gland interfaces, enabling dissection along anatomical planes that preserve normal tissue. In addition, achieving GTR usually indicates that the surgical field was well visualized with angled endoscopy and that tumor removal was performed in a controlled fashion with careful preservation of the gland and stalk. Therefore, GTR may function as a composite marker of both biologically less aggressive tumors and optimal surgical exposure and technique. Other series have similarly shown that tumors amenable to GTR tend to have better global outcomes, including visual and endocrine results, than tumors in which only subtotal resection is feasible.^[13]^

Long-term systemic glucocorticoid therapy and indicators of surgical complexity (longer operation time, higher blood loss, intraoperative CSF leak) were significant or borderline factors in univariate analyses but did not remain independent predictors after adjustment. The attenuation of these associations suggests that their effects are largely mediated through tumor size and invasiveness. Glucocorticoid exposure may mask subtle preoperative hypothalamic–pituitary–adrenal axis dysfunction and complicate early postoperative assessment, but in this cohort, it did not independently predict persistent hypopituitarism after accounting for anatomical and surgical factors. Similarly, operation time and blood loss likely reflect the underlying complexity imposed by large, invasive tumors rather than serving as direct determinants of endocrine injury. Preoperative clinical features, including sex, BMI, and common systemic comorbidities, were not significantly associated with postoperative hypopituitarism. Although age was higher in the hypopituitarism group, it did not remain an independent factor in the multivariable model, indicating that age-related vulnerability may be overshadowed by tumor-related factors when these are considered simultaneously. Preexisting any-axis hypopituitarism also did not predict further postoperative deterioration in this analysis, which may be related to sample size and to the composite definition of the endpoint. Nevertheless, the relatively high baseline prevalence of axis deficits underscores the need for systematic endocrine assessment both before and after surgery.^[14]^

The present findings align with and extend recent literature on endocrine outcomes after transsphenoidal surgery in the endoscopic era. Several contemporary series have reported that tumor volume and invasiveness are key determinants of postoperative pituitary function. A 2022 study on endoscopic surgery for PitNETs reported that larger tumor volume and higher Knosp grade were associated with adverse surgical outcomes, including more frequent non-GTR and less favorable endocrine recovery. Galal et al similarly found that preoperative panhypopituitarism and large, invasive tumors were associated with poor postoperative hormonal recovery, emphasizing the interaction between tumor burden and surgical complexity.^[15]^ More recently, Galal et al analyzed predictors of hormone replacement after endoscopic resection and showed that tumor size, cavernous sinus invasion, and extent of resection significantly influenced postoperative endocrine outcomes, consistent with the present observation that tumor diameter and Knosp grade 3 to 4 independently increased the risk of hypopituitarism.^[15]^ Regarding the extent of resection, prior work has mainly focused on oncologic and visual endpoints, with GTR associated with lower recurrence and better visual outcomes, but its relationship with endocrine function has been less consistent.^[4,16]^ Some studies have reported no clear association between GTR and new-onset hypopituitarism, possibly due to heterogeneity in surgical techniques or inclusion of microscopic procedures. In contrast, Pala et al observed a low rate of new hypopituitarism, predominantly in patients undergoing microscopic rather than endoscopic surgery, suggesting that endoscopic visualization may facilitate both more complete and safer resections.^[17]^ In the current cohort, GTR emerged as an independent protective factor, supporting the concept that, in experienced hands and carefully selected cases, complete resection and preservation of pituitary function are not mutually exclusive goals.^[18]^

Several limitations should be acknowledged. First, the retrospective, single-center design is subject to selection bias and limits generalizability to centers with different surgical experience or endocrine protocols. Second, the number of hypopituitarism events was modest, which may limit statistical power and increase the risk of model overfitting. Third, variables such as tumor consistency, detailed histological subtype, proliferation markers, and intraoperative perfusion-related parameters were not systematically available. Fourth, the study focused on early postoperative endocrine status within the predefined early follow-up window; therefore, the findings should not be extrapolated to long-term pituitary function, late recovery, or delayed deterioration. Finally, the composite endpoint did not distinguish axis-specific patterns of vulnerability. Future multicenter prospective studies with longer follow-up, standardized hormonal testing schedules, and integration of histopathologic and molecular markers are needed to validate these findings.

## 5. Conclusion

In patients with PitNETs undergoing EETS, larger tumor size and higher Knosp grade independently increased the likelihood of early postoperative hypopituitarism, whereas GTR was associated with reduced risk. These findings indicate that tumor anatomy and extent of resection are key determinants of early endocrine outcomes and should inform preoperative counseling, surgical planning, and early postoperative surveillance. Longer-term follow-up is required to determine whether these early risk factors also predict sustained pituitary recovery or delayed endocrine deterioration.

## Author contributions

**Conceptualization:** Li Zhang, Qiuyan Gao, Wei Dong, Yu Zhang, Xiao Liu Dong.

**Data curation:** Li Zhang, Qiuyan Gao, Wei Dong, Yu Zhang, Xiao Liu Dong.

**Formal analysis:** Li Zhang, Qiuyan Gao, Wei Dong, Yu Zhang, Xiao Liu Dong.

**Funding acquisition:** Li Zhang, Xiao Liu Dong.

**Writing – original draft:** Li Zhang, Xiao Liu Dong.

**Writing – review & editing:** Li Zhang, Xiao Liu Dong.

**Investigation:** Xiao Liu Dong.
